# Are the functions of non-suicidal self-injury associated with its persistence and suicide risk in university students? Insights from a network analysis

**DOI:** 10.3389/fpsyt.2024.1442930

**Published:** 2024-11-11

**Authors:** Monika Szewczuk-Bogusławska, Krzysztof Kowalski, Bogna Bogudzińska, Błażej Misiak

**Affiliations:** Department of Psychiatry, Wroclaw Medical University, Wroclaw, Poland

**Keywords:** self-harm, suicidality, depression, early intervention, dissociation

## Abstract

**Background:**

To date, a number of intra- and interpersonal functions of non-suicidal self-injury (NSSI) have been identified. Yet, their association with persistence of NSSI and suicide risk remains unknown. The study aimed to investigate which functions of NSSI are associated with its persistence and suicide risk in university students using a network analysis.

**Methods:**

Altogether, 830 university students reporting a lifetime history of NSSI were enrolled. The persistence of NSSI was defined as its presence over preceding 12 months.

**Results:**

Persistent NSSI was directly connected to the nodes representing two functions of NSSI: affect regulation and self-punishment. Suicide risk was directly connected to the anti-suicide function of NSSI. The shortest pathway from persistent NSSI to suicide risk led through depressive symptoms. Other likely pathways (three mediating nodes) led through the functions of NSSI (affect regulation or self-punishment, anti-dissociation, and anti-suicide). Depressive symptoms had the highest centrality. However, it did not differ significantly compared to some functions of NSSI (marking distress, anti-dissociation, toughness, and affect regulation).

**Discussion:**

In university students, persistent NSSI might be directly associated with its functions related to affect regulation and self-punishment, while suicide risk might be directly associated with the anti-suicide function. The observations posit a role of intrapersonal functions in shaping the outcomes of NSSI. Depressive symptoms and some NSSI functions might be the most promising targets for interventions in this population.

## 1 Introduction

The transition to adulthood is an important life period accompanied by approaching new roles and responsibilities. In developed countries, the majority of adolescents decide to continue education (1). Undergraduate university students often face with stressful situations related to academic pressure, economic constraints, and new social relationships (2). Exposure to these stressors in vulnerable individuals might contribute to the emergence of mental health crisis. A recent meta-analysis estimated the pooled prevalence of depression or depressive symptoms at 25%. In turn, the prevalence of suicide-related outcomes was found to be 14% (3). Although suicide rates seem to be lower in university students compared to the general populations, there is evidence that they tend to increase in recent years (4, 5).

To date, several risk factors of suicide have been identified. Among them, previous suicide attempts and ideation are still considered the strongest risk factors (6). However, self-harming behaviors may also emerge without an explicit suicidal intent. This type of behaviors, defined as a deliberate destruction of body parts without a suicidal intent and for purposes not socially sanctioned, is commonly referred to as non-suicidal self-injury (NSSI) (7). Importantly, NSSI is also perceived as a risk factor of suicide (8, 9). Although NSSI has been recognized as a suicide risk factor, its frequency and persistence over time, the impact on daily functioning, and functions are crucial for clinical assessment and prognosis (10).

Among several characteristics of NSSI, assessment of its functions might hold the potential for planning specific interventions (11). revealed a two-factor structure of NSSI functions, i.e., interpersonal and intra-personal ones. Interpersonal functions reflect specific motivations underlying NSSI that appear to influence others for various purposes (e.g., to express one’s own autonomy and demonstrate a lack of intent to seek help, to draw others attention to physical pain, and to take revenge on someone) (12). Contrary to the social motivation of interpersonal functions, intra-personal functions serve as coping strategies to alleviate, reduce, or eliminate difficult inner emotions, feelings, or thoughts. They are represented by affect regulation, anti-dissociation, anti-suicide, self-care, self-punishment and marking distress functions. However, these effects, although maladaptive, appear to be effective in a short-term perspective (13). There is evidence that individuals who engage in NSSI endorse multiple functions; however, intra-personal functions are more frequently endorsed (14).

It has been found that the functions of NSSI might be differentially associated with co-occurring psychopathology. Specifically, it has been shown that intra-personal functions, but not interpersonal ones, might be associated with hopelessness, post-traumatic stress disorder symptoms, and a higher suicide risk (15, 16). Similarly, it has been found that intra-personal functions of NSSI might predict its persistence over time (17). However, it has also been shown that intra-personal functions of NSSI might be stronger predictors of its persistence during hospitalization than after discharge (18). Of note, individuals with persistent NSSI compared to those with recovered NSSI have been found to show significantly higher suicide risk (19). There is also some evidence that the intrapersonal functions moderate both the effects of NSSI frequency and methods on suicide risk while the interpersonal functions moderate the effects of NSSI frequency on suicide risk among undergraduate students (20).

Previous studies have not investigated as to whether specific functions of persistent NSSI are associated with suicide risk. To address this research gap, the present study aimed to investigate which functions of NSSI bridge its persistence with suicide risk. Due to the complexity of intra-personal and interpersonal functions and potential effects of co-occurring psychopathology, we decided to address this aim using a network analysis (21). This approach enables to analyze multiple variables in a single model without a predefined causality (22). Moreover, a network analysis allows to indicate the most central variables in the network, i.e., those that show the greatest strength of connections with other variables (23). According to a network theory, these variables should be considered the most promising targets for interventions. This is due to the fact that reducing their level is likely to decrease the spread of information between all variables in the network.

## 2 Materials and methods

### 2.1 Sampling procedures

Participants were students of universities located in two academic cities in Poland, i.e., Wroclaw (about 108,000 university students) and Opole (about 15,800 university students). They were recruited in frame of an ongoing campaign “*Talk about yourself*” that aims to open the academic society towards concerns related to mental health of university students and inform about possibilities of approaching mental health care in case of experienced crisis. The campaign was launched in October, 2023. The sample reported in the present study was developed between October, 2023 and January, 2024.

Students were informed about the campaign through the websites of universities, emails, social media, radio and television interviews with mental health professionals, and flyers disseminated in the academic society. Simultaneously, they were invited to participate in the anonymous internet-based survey. The survey included questions about sociodemographic characteristics, a history of psychiatric treatment, psychopathological symptoms, and a history of NSSI. All individuals have provided consent to participate in the study. The study protocol was approved by the Bioethics Committee at Wroclaw Medical University, Wroclaw, Poland (approval number: 197/2023N).

### 2.2 Measures

#### 2.2.1 NSSI and its functions

To characterize a history of NSSI, we used the following questions with yes-or-no responses: 1) “Have you ever intentionally injured your body without the intent to commit suicide?”; 2) “How old were you when you did it for the first time? and 3) “Did you intentionally injured your body without the intent to commit suicide in preceding 12 months?”. Individuals with a positive response to the third question were further asked about the frequency of thinking about NSSI (“How often do you think about injuring intentionally your body without the intent to commit suicide?”, possible responses were: “almost all the time”, “everyday”, “once a week”, “once a month”, and “almost not at all”).

To record the functions of NSSI, we used the Inventory of Statements about Self-Injury (ISAS) (12). It is a self-report that includes 39 items about the reasons to engage in NSSI. Each function of NSSI is recorded by three items rated on a three-point scale (0 – “not relevant”, 1 – “somewhat relevant”, and 2 – “very relevant”). Altogether 13 functions are measured: 1) affect regulation; 2) interpersonal boundaries; 3) self-punishment; 4) self-care; 5) anti-dissociation; 6) anti-suicide; 7) sensation seeking; 8) peer bonding; 9) interpersonal influence; 10) toughness; 11) marking distress; 12) revenge and 13) autonomy. A higher score obtained for a specific function of NSSI indicates its greater endorsement.

#### 2.2.2 Depressive symptoms

Depressive symptoms were measured using the Patient Health Questionnaire-8 (PHQ-8) (24). The PHQ-8 records the presence of depressive symptoms over the period of preceding 2 weeks. The items are rated on a three-point scale (responses range from 0 – “not at all” to 3 – “nearly every day”). In this regard, the total PHQ-8 score ranges between 0 and 24. Higher scores are equivalent to a greater severity of depressive symptoms. In the present study, the Cronbach’s alpha for the PHQ-8 was 0.817.

#### 2.2.3 Anxiety symptoms

Anxiety symptoms were assessed using the Generalized Anxiety Disorder-7 (GAD-7) (25). The GAD-7 records the presence of anxiety symptoms over the period of preceding 2 weeks. The items are rated on a three-point scale (responses range from 0 – “not at all” to 3 – “nearly every day”). Therefore, the total GAD-7 score ranges between 0 and 21. Higher scores are equivalent to a greater level of anxiety symptoms. In the present study, the Cronbach’s alpha for the GAD-7 was 0.910.

#### 2.2.4 Psychotic-like experiences

Psychotic-like experiences were measured using the Prodromal Questionnaire-16 (PQ-16) (26). The PQ-16 was developed to screen for the presence of clinical high risk of psychosis. The questionnaire is based on two subscales. The first subscale records the presence of psychotic-like experiences using true-or-false responses (rated as 1 or 0) while the second one is focused on associated distress. In our study, we did not include the second subscale. Two items might conceptualize the presence of depressive and anxiety symptoms (item 1: “I feel uninterested in things I used to enjoy” and item 7: “I get extremely anxious when meeting people for the first time”). To avoid the overlap of these items with those from the PHQ-8 and GAD-7, they were excluded from calculating the total score. In this regard, the total score on the questionnaire measuring psychotic-like experiences ranged between 0 and 14. Higher scores are equivalent to a greater level of psychotic-like experiences. The Cronbach’s alpha in the present study was 0.787.

#### 2.2.5 Dissociation

Dissociation symptoms were determined using the revised version of the Dissociation Experiences Scale (DES) – Taxon (DES-T) (27). The DES-T includes eight items from the original DES that were reported to be the most predictive for the occurrence of dissociative disorders (28). To improve clinical usefulness of the DES-T, its revised version has been proposed. In this version, a novel rating system has been proposed (from 0 – “never” to 7 – “once a day or more”) (27). One item of the revised DES-T refers to auditory hallucinations (“some people sometimes find that they hear voices inside their head which tell them to do things or comment on things that they are doing”). To avoid the overlap of dissociation symptoms with psychotic-like experiences, this item was excluded from calculating the total DES-T score. Therefore, in the present study the total DES-T score ranged between 0 and 56. The Cronbach’s alpha was 0.824 in our sample.

#### 2.2.6 Insomnia

The presence of insomnia was measured using the Insomnia Severity Index (ISI) (29). It is a brief, seven-item measure of insomnia over the period of preceding two weeks. Three items refer to the type of insomnia problem (difficulty falling asleep, difficulty staying asleep, and problems waking up too early). Other items measure satisfaction with current sleep pattern, noticeability of sleep problem to others, worry (distress) about the current sleep problem, and interference of the sleep problem with daily functioning. All items are rated on four-point scale. The total ISI score ranges between 0 and 28. Higher scores are equivalent to a greater level of insomnia. The Cronbach’s alpha was 0.840 in our sample.

#### 2.2.7 Suicide risk

The risk of suicide was assessed with the suicidality section of the Mini-International Neuropsychiatric Interview (M.I.N.I.) (30). It is based on five questions referring to the preceding month: “did you think that you would be better off dead or wish you were dead?” (score: 1 point), “did you want to harm yourself or to hurt or injure yourself?” (score: 2 points), “did you think about suicide?” (score: 6 points), “did you have a suicide plan?” (score: 10 points), and “did you attempt suicide?” (score: 10 points). In addition, one question measures a lifetime history of suicide attempt (“in your lifetime, did you ever make a suicide attempt?”, score: 4 points). The risk of suicide is calculated as the sum of points across the whole section (range: 0 – 33). Higher scores indicate a greater risk of suicide. The Cronbach’s alpha in the present study was 0.707.

### 2.3 Data analysis

The data analysis was limited to participants reporting a positive lifetime history of NSSI. They were divided into those with a positive history of NSSI over the period of preceding 12 months (persistent NSSI) and those with a negative history of NSSI in this period (non-persistent NSSI). In the first step of data analysis, these groups of participants were compared using the χ^2^ test (categorical variables) and the Mann-Whitney U test or t-tests (depending on data distribution). This part of data analysis was carried out in the SPSS software, version 28. Results were interpreted as significant if the p-value was < 0.05.

Next, we performed a network analysis in the R software (version 4.1.3, see **Supplementary Appendix** for the R code) that included the following groups of variables: the persistence of NSSI, functions of NSSI, suicide risk, and covariates (age, female gender vs. other gender identifications, a lifetime history of psychiatric treatment, and the scores of psychopathological symptoms). As the data included both binary (the persistence of NSSI, gender, and a lifetime history of psychiatric treatment) and continuous variables (functions of NSSI, suicide risk, and the measures of psychopathology), we used the mixed graphical models (the *mgm* package) to estimate the network (31). To improve the prediction accuracy and interpretability of the network, the L1-penalized regression (LASSO) was used (32). The LASSO shrinks partial correlation coefficients, thereby small coefficients are converted to zero. This allows to avoid indicating spurious and weak associations. In this regard, all visualized edges refer to significant connections. The Extended Bayesian Information Criterion (EBIC), using the tuning parameter λ that controls the level of sparsity, was implemented to select the penalty parameter (33). The λ parameter of 0.5 was used as proposed previously (32). The resulting network presents variables as nodes that are linked with edges. Thicker edges correspond with greater partial correlation coefficients.

To assess the node centrality, we estimated the node strength. It can be defined as the sum of weights for edges directly connected to a specific node (23, 34). Also, the predictability (i.e., the proportion of variance explained by all edges linked to a specific node) was calculated for each node. All visualizations were performed using the *qgraph* package (35, 36).

The final part of a network analysis was related to assessment of stability and accuracy in the *bootnet* package (34). Stability of the node strength was assessed following the case-drop bootstrapping procedure with 1,000 iterations. In turn, the non-parametric bootstrapping with 1,000 iterations was carried out to assess stability of edge weights. The network was considered stable if the correlation stability coefficient (CS-C) was higher than 0.25.

## 3 Results

### 3.1 The general characteristics of the sample

A total of 1653 university students were surveyed (**Table 1**). Among them, 830 university students reported a lifetime history of NSSI (50.2%). Persistent NSSI was reported by 450 university students (27.2% of the initial sample). The frequency of thinking about NSSI was once a month or higher in 71.5% of university students with persistent NSSI. Students with persistent NSSI were significantly younger, reported significantly higher rates of lifetime psychiatric treatment and psychotherapy as well as they were significantly less likely to be employed. Also, both groups differed significantly with respect to gender (i.e., a higher percentage of women, a lower percentage of men, and higher number of individuals reporting other gender identities among those with persistent NSSI). Psychopathological symptoms had a significantly higher severity in students with persistent NSSI across all recorded domains. Both groups also differed significantly with respect to functions of NSSI, i.e., affect regulation, interpersonal boundaries, self-punishment, self-care, anti-dissociation, anti-suicide, peer bonding marking distress, and autonomy. All of these functions, except for peer bonding, were significantly more likely to be endorsed by students with persistent NSSI.

**Table 1:** Descriptive characteristics of the sample.

	Persistent NSSI (*n* = 450)	Non-persistent NSSI (*n* = 380)	*p*
Age, years	21.0 (2.3)	21.7 (2.7)	< 0.001
Gender			
Women Men Other	363 (80.7%) 61 (13.6%) 26 (5.7%)	301 (79.2%) 72 (18.9%) 7 (1.9%)	0.003
Current employment, yes	182 (40.4%)	185 (48.7%)	0.017
Lifetime history of psychiatric treatment and psychotherapy, yes	354 (78.7%)	247 (65.0%)	< 0.001
NSSI – age of onset, years	14.7 (3.3)	14.1 (2.3)	0.132
Current frequency of thinking about NSSI			
Almost all the time Everyday Once a week Once a month Almost not at all	15 (3.3%) 74 (16.4%) 111 (24.7%) 122 (27.1%) 128 (28.5%)	–	–
NSSI – function			
Affect regulation Interpersonal boundaries Self-punishment Self-care Anti-dissociation Anti-suicide Sensation seeking Peer bonding Interpersonal influence Toughness Marking distress Revenge Autonomy	4.7 (1.5) 0.5 (1.1) 3.7 (2.0) 1.3 (1.4) 2.4 (1.9) 1.9 (2.0) 0.8 (1.2) 0.09 (0.52) 0.8 (1.3) 1.2 (1.5) 2.6 (2.0) 0.3 (0.9) 0.8 (1.4)	3.3 (2.0) 0.3 (0.8) 2.7 (2.2) 1.0 (1.4) 1.8 (1.9) 1.2 (1.6) 0.7 (1.1) 0.14 (0.56) 0.8 (1.3) 1.1 (1.4) 2.1 (2.0) 0.3 (0.9) 0.4 (1.1)	0.001 < 0.001 < 0.001 < 0.001 < 0.001 < 0.001 0.167 0.029 0.615 0.457 < 0.001 0.991 < 0.001
M.I.N.I., suicide risk	13.8 (11.3)	9.0 (9.7)	< 0.001
PHQ-8, depressive symptoms	15.9 (4.4)	13.2 (4.7)	0.001
GAD-7, anxiety symptoms	13.2 (5.5)	11.2 (5.5)	< 0.001
DES-T, dissociation symptoms	11.1 (9.0)	7.1 (7.1)	< 0.001
PQ-16, psychotic-like experiences	6.6 (3.3)	5.2 (3.1)	< 0.001
ISI, insomnia	11.8 (3.8)	10.5 (5.6)	0.003

DES-T, the Dissociation Experiences Scale – Taxon (DES-T); GAD-7, the Generalized Anxiety Disorder-7; ISI, the Insomnia Severity Index; M.I.N.I., the Mini-International Neuropsychiatric Interview; NSSI, non-suicidal self-injury; PHQ-8, the Patient Health Questionnaire-8; PQ-16, the Prodromal Questionnaire-16.

Data expressed as n (%) or mean (SD).

### 3.2 The network

**Figure 1:**
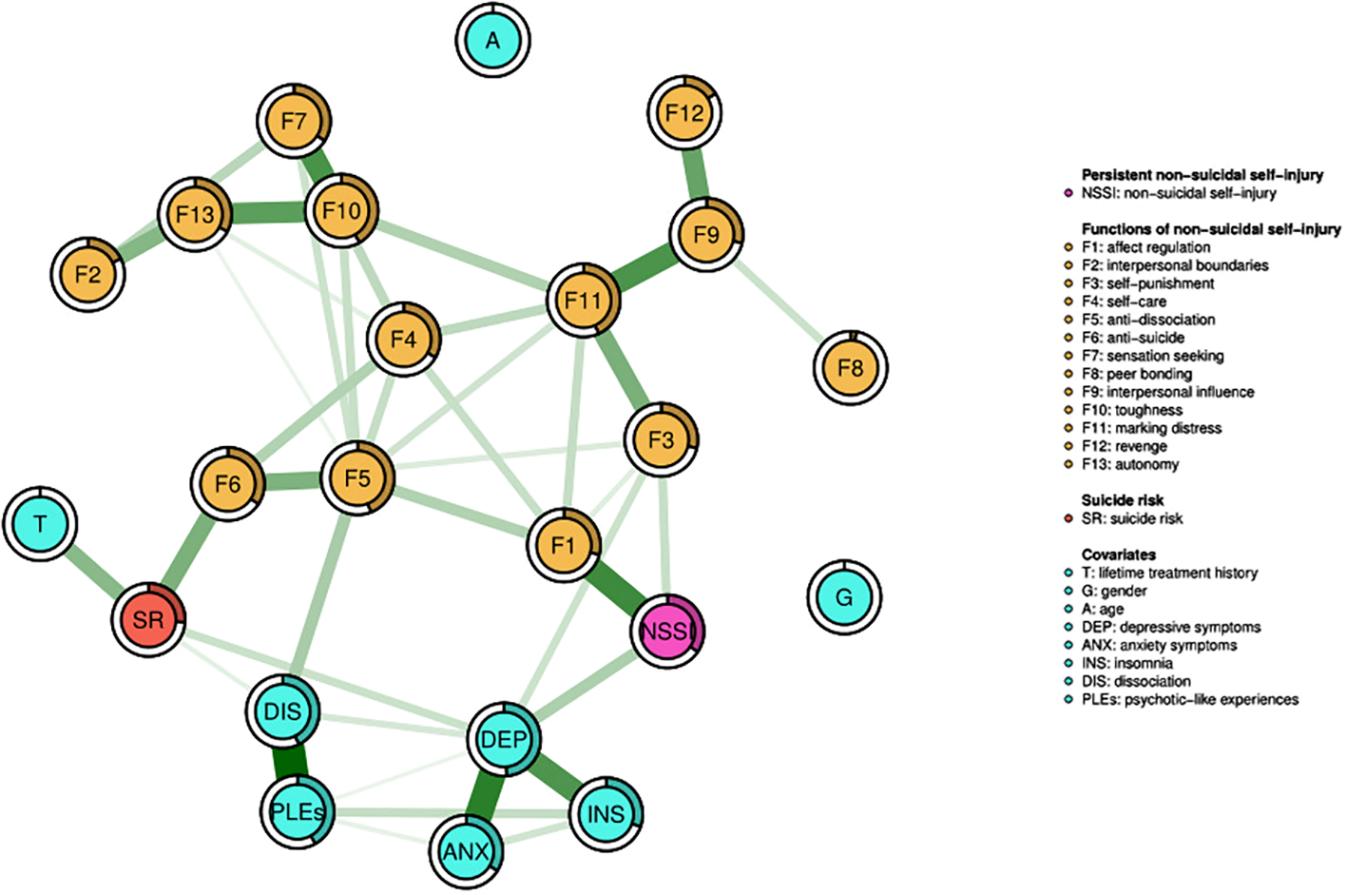
The network analyzed in the present study. It shows variables that are presented as nodes that are connected with edges. All edges refer to significant and positive associations. Thicker edges are equivalent to stronger associations. The filled part of the ring around each node presents the node predictability.

The network analyzed in the present study is shown in **Figure 1**. Almost all nodes appeared to be well-connected and no negative edges were observed. The nodes representing age and gender were not connected to any other nodes in the network. Among 253 potential edges, 41 partial correlation coefficients were found to have a non-zero value (16.2%, **Supplementary Table 1**).

The node representing persistent NSSI was directly connected to the nodes for two NSSI functions, i.e., affect regulation (weight = 0.322) and self-punishment (weight = 0.099). The difference in these edge weights was significant (**Supplementary Figure 1**). In turn, suicide risk was directly connected to the nodes representing the anti-suicide function of NSSI (weight = 0.220), a lifetime history of psychiatric treatment and/or psychotherapy (weight = 0.196), depressive symptoms (weight = 0.092), and dissociation symptoms (weight = 0.047). Among them, the edge weights for connections of suicide risk with the anti-suicide function of NSSI and a lifetime history of psychiatric treatment and/or psychotherapy were significantly higher compared to other edge weights (**Supplementary Figure 1**). However, the most likely pathway from persistent NSSI to suicide risk led through depressive symptoms. Alternative pathways included those through affect regulation or self-punishment, anti-dissociation, and anti-suicide functions of NSSI.

### 3.3 Node centrality and predictability

**Figure 2:**
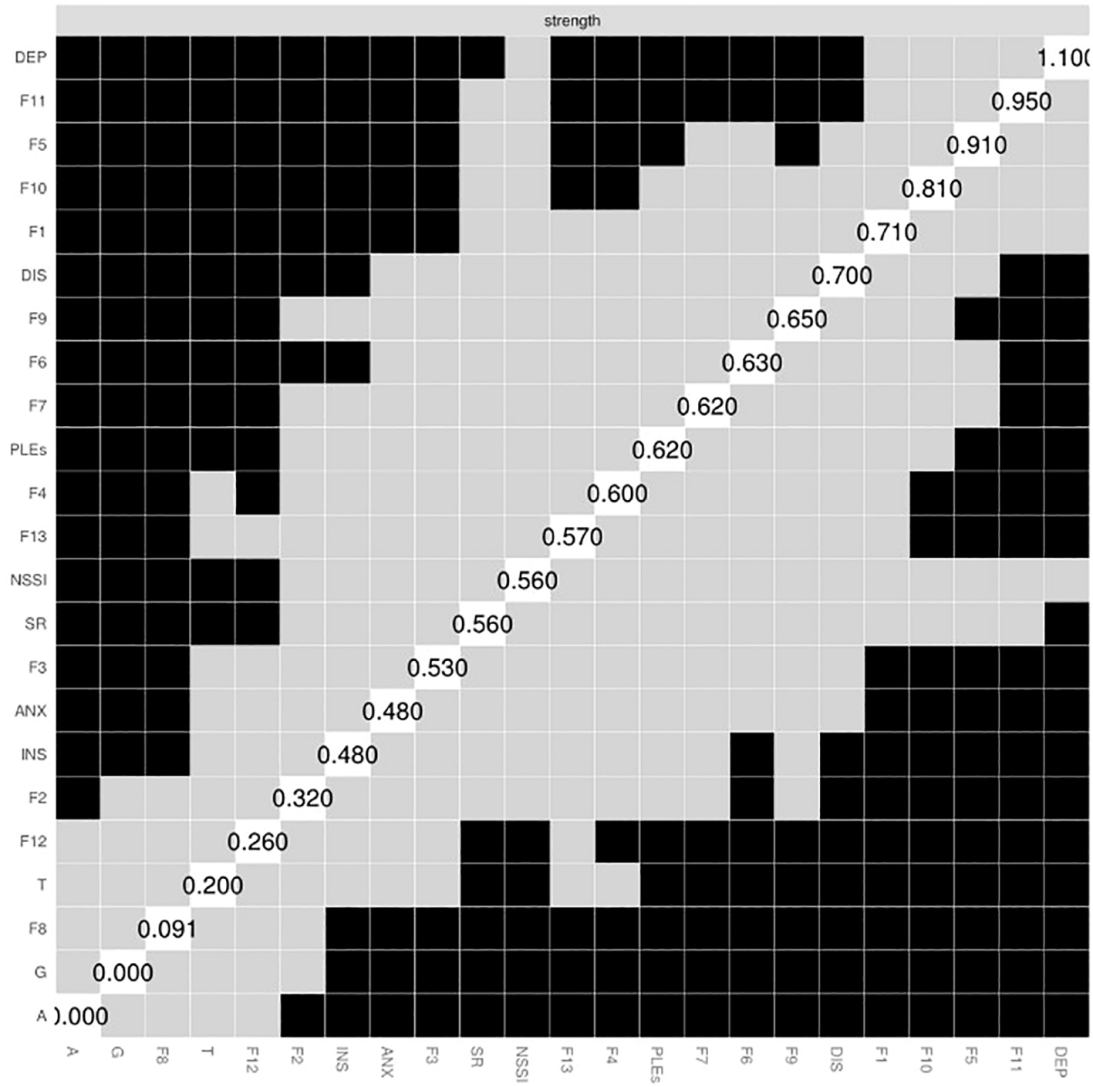
The matrix of strength centrality indices. Black boxes illustrate significant between-node differences in the edge centrality. A, age; ANX, anxiety symptoms; DEP, depressive symptoms; DIS, dissociation symptoms; F1, affect regulation; F2, interpersonal boundaries; F3; self-punishment; F4, self-care; F5, anti-dissociation; F6, anti-suicide; F7, sensation seeking; F8, peer bonding; F9, interpersonal influence; F10, toughness; F11, marking distress; F12, revenge; F13, autonomy; G, gender; INS, insomnia; NSSI, persistent non-suicidal self-injury; PLEs, psychotic-like experiences; SR, suicide risk.

Depressive symptoms had the highest strength centrality in the network (**Figure 2**). However, the strength centrality of depressive symptoms did not differ significantly compared to the strength centrality of the nodes representing four functions of NSSI, i.e., marking distress, anti-dissociation, toughness, and affect regulation.

The average predictability of nodes in the network was 0.281 (**Supplementary Table 2**). The highest predictability was observed for depressive symptoms (0.489), followed by the anti-dissociation (0.441) and marking distress (0.433) functions of NSSI, dissociation symptoms (0.427), and psychotic-like experiences (0.420).

### 3.4 Network stability and accuracy

**Supplementary Figures 2**, **3** show stability of the strength centrality and edge weights. The CS-C values for the node strength and edge weights were 0.672 and 0.749, respectively. These statistics indicate that results of the network analysis were stable while dropping various proportions of data. Also, the 95%CI of edge weights after bootstrapping was relatively narrow supporting the network accuracy.

## 4 Discussion

### 4.1 Main findings

Findings from the present study indicate that depressive symptoms and two functions of NSSI, i.e., affect regulation and self-punishment, are directly associated with its persistence. The affect-regulation function of NSSI can be defined as the one that aims to “alleviate acute negative affect or aversive affective arousal” (37). To understand the mechanisms of the affect-regulation function of NSSI, some theories posit that an early social disadvantage can teach individuals to use maladaptive strategies for coping with negative emotions (38). Notably, affect or emotion regulation function has been found among the most frequent functions of NSSI (39). Difficulties in emotion regulation have also been reported to underly a low desire to cease NSSI (40). In turn, the self-punishment function refers to using NSSI as the way of expressing anger towards oneself. Self-punishment, most commonly for the reasons related to “being bad or having bad thoughts”, might be ranked as the second most commonly endorsed function of NSSI (37). Both self-punishment and affect regulation represent intra-personal functions of NSSI that have collectively been associated with persistence of NSSI over time (41, 42).

Notably, our study provides insights into the functions of NSSI underlying its association with suicide risk. Specifically, our network analysis revealed that the shortest pathway (i.e., through a mediating effect of one node) from persistent NSSI leads through depressive symptoms. However, alternative pathways were also possible. These pathways appeared to be activated by self-punishment and affect-regulation functions leading through the anti-dissociation and anti-suicide function of NSSI (i.e., three mediating nodes). The later one was directly connected to suicide risk. This observation is consistent with the prior results showing that engagement in NSSI to stop suicidal thoughts or avoid suicide attempt is finally associated with a higher suicide risk than using NSSI for other reasons (43, 44). Paul, Tsypes (44) analyzed the functions of NSSI in university students and found the relationships between suicide attempts and nearly every function of NSSI, with the strongest risk driven by anti-suicide, anti-dissociation, and coping with self-hatred functions. In turn, Brausch and Muehlenkamp (43) revealed that the perceived effectiveness of NSSI for anti-suicide effect predicts planning and attempting suicide. Moreover, the authors observed that the expectation for the NSSI effectiveness of self-punishment predicts suicidal ideations, plans, and attempts.

The analysis of centrality metrics also provides important insights for clinical practice. Although the highest strength centrality was found for depressive symptoms, it was comparable to the strength centrality metrics of some NSSI functions, i.e., marking distress, anti-dissociation, toughness, and affect regulation. Following the accounts of a network theory, the most central nodes are potential candidates for interventions as reducing their level might result in decreased spread of information within the network. In this regard, our observations suggest that depressive symptoms, intra-personal (anti-dissociation and affect regulation) and interpersonal functions (marking distress and toughness) need to be considered when planning the therapy of individuals with persistent NSSI. As discussed above, anti-dissociation and affect regulation functions are important correlates of NSSI persistence and NSSI-related suicide risk. Altogether these observations support the rationale to personalize the therapeutic plan for individuals with NSSI according to its functions. For instance, the dialectical behavior therapy (45) or emotional regulation group therapy (ERGT) (46) are often used in people with NSSI; however, they are focused on maladaptive emotion regulation strategies. In this regard, these approaches may not show a desired efficacy in the therapy of NSSI for individuals who do not endorse the affect-regulation function of NSSI. Another example is the compassion-focused therapy that might be an optimal option for individuals who use NSSI as the way of self-punishment (47).

The findings might also be important in the context of emerging adulthood and developmental goals of university students. A recent analysis of data from the World Mental Health College Student (WMH-ICS) initiative estimated the lifetime and 12-month prevalence of NSSI in first-year college students at 17.7% and 8.4%, respectively (48). Moreover, this analysis revealed that NSSI appears in various mental disorders representing a behavioral marker of psychopathology. Important findings were also reported by a longitudinal study focused on college entrants (data from the Leuven College Surveys). The authors found that a one-year incidence of first-onset NSSI was 10.3% in during the first year and 7.0% during the second year. A history of violence before the age of 17 and severe impairment of social functioning were observed to be the strongest predictors of NSSI onset (49). It is also important to note that college entrants with NSSI are at risk of poor academic performance. Overall stress and test anxiety have been indicated as potential mechanisms linking NSSI and academic performance (50). Taking into account the impact of NSSI on university students, our findings might further inform the development of interventions in this population. Specifically, the observation that specific functions of NSSI are associated with its persistence (affect regulation and self-punishment functions) and suicide risk (anti-dissociation function) posit the necessity to include this aspect while planning intervention. At this point, it is further important to note interindividual variability of potential mechanisms underlying NSSI in university students. For instance, the study by Christoforou et al. (51) demonstrated the presence of three latent emotional profiles among university students reporting NSSI including students with considerable emotion difficulties, those with passive moderate emotion difficulties, and those without emotion difficulties. The profiles appeared to have similar frequencies. These observations suggest that the interventions targeting NSSI in university students need to be personalized taking into consideration underlying psychological mechanisms. Another mechanism underlying lower academic performance among individuals with NSSI is related to cognitive impairments. Indeed, it has been shown that individuals with NSSI present worse performance of information processing speed, working memory attention/alertness, visual learning, speech solving, and reasoning (52).

### 4.2 Limitations

Certain limitations of this study need to be discussed. First, the sample was not large and *a priori* power calculations were not carried out. However, there is no general consensus for estimating required sample size for analyses based on mixed graphical models. It should also be noted that the results were stable while dropping various proportions of data. Second, representativeness of the sample might be limited as we were not able to record the initial number of students approached for participation. In this regard, the reasons of non-participation cannot be indicated. Also, as the survey was limited to university students, the potential to generalize findings to other populations is limited. Third, internet-based surveys may provide limited data accuracy. However, this approach, due to confidentiality issues, might provide opportunities to disclose information about sensitive data related to mental health. Fourth, the study was based on self-reports and lacked a thorough in-person clinical assessment. Therefore, we were unable to assess a psychiatric diagnosis in our sample. Fifth, the participants classified as those showing persistent NSSI were significantly younger. It should be noted that the prevalence of NSSI tends to decrease with age. However, in the network analysis, the node representing age appeared to be isolated, i.e., it was not connected to any variable in the network. Sixth, a one-year remission from NSSI may not exclude the possibility of its recurrence. Finally, the study did not have a longitudinal design and thus causality should be interpreted with caution.

### 4.3 Conclusions and implications

Altogether, results from the present study highlight the need to assess functions of NSSI during clinical assessment. Intra-personal functions including affect regulation, self-punishment, anti-dissociation, and anti-suicide functions might be associated with persistence of NSSI and suicide risk related to NSSI in university students. Interventions targeting these functions, as well as some interpersonal functions (marking distress and toughness) together with depressive symptoms might hold promise for the discontinuation of NSSI and reduction of NSSI-related suicide risk. Assessment of NSSI functions should be highlighted as an important aspect of future studies that aim to develop and use personalized therapeutic interventions. This is of importance as various interventions might hold a differential efficacy in people endorsing specific functions of NSSI. However, longitudinal studies addressing the effects of functions underlying persistent NSSI on suicide risk are still needed to provide evidence of causality.

## Data Availability

The raw data supporting the conclusions of this article will be made available by the authors, without undue reservation.
