# Supplementary material for: Are the functions of non-suicidal self-injury associated with its persistence and suicide risk in university students? Insights from a network analysis
Source: Front Psychiatry. 2024 Nov 11;15:1442930. doi: 10.3389/fpsyt.2024.1442930 (PMC11586333; doi:10.3389/fpsyt.2024.1442930)

**Supplementary Appendix**

**The R code used in data analysis**

####Loading required packages#####

library(qgraph)

library(bootnet)

library(mgm)

library(OpenMx)

library(networktools)

library(NetworkComparisonTest)

####Loading the database####

setwd("…")

database <- read.csv2("database.csv", sep = ';', header=TRUE)

View(database)

data <- as.matrix(database)

data[is.na(data)] <- 0

####Defining variables of interest####

type <- c(rep("c", 8), rep("g", 9))

level <- c(2, 1, 1, 1, 1, 1, 1, 1, 1, 1, 1, 1, 1, 1, 1, 2, 2, 1, 1, 1, 1, 1, 1)

labels <- c("NSSI", "F1", "F2", "F3", "F4", "F5", "F6", "F7", "F8", "F9", "F10", "F11", "F12", "F13", "SR", "T", "G", "A", "DEP", "ANX", "INS", "DIS", "PLEs")

names <- c("non-suicidal self-injury", "affect regulation", "interpersonal boundaries", "self-punishment", "self-care", "anti-dissociation", "anti-suicide", "sensation seeking", "peer bonding", "interpersonal influence", "toughness", "marking distress", "revenge", "autonomy", "suicide risk", "lifetime treatment history", "gender", "age", "depressive symptoms", "anxiety symptoms", "insomnia", "dissociation", "psychotic-like experiences")

grouplabels <- list("Persistent non-suicidal self-injury" = c(1:1), "Functions of non-suicidal self-injury" = c(2:14), "Suicide risk" = c(15:15), "Covariates" = c(16:23)

group_cols <- c("#ba60bd", "#faa43a", "#f15854,"#50d9d2")

####Network estimation####

Fit <- mgm(data = data, type = type, level = level, labels = labels, lambdaSel = "EBIC", lambdaGam = 0.5, scale = TRUE, binarySign = TRUE)

net <- qgraph(Fit$pairwise$wadj, vTrans = 200, layout = 'spring', labels = labels, nodeNames = names, border.width=1.5, border.color="black", groups = grouplabels, color = group_cols, legend.cex=.40, label.cex = 1.2, GLratio = 1.8, vsize = 5, esize = 16, posCol = "darkblue", negCol = "red", cut = 0)

####Edge weights####

myedges <-getWmat(net)

write.csv(myedges, "MyNetworkEdges.csv")

####Node predictability####

pred <- predict(Fit, data)

pred$errors

pie1a <- as.numeric(as.character(pred$errors[c(2:15, 18:13), 3]))

pie1b <- as.numeric(as.character(pred$errors[c(1, 16:17), 5]))

pie1 <- c(pie1a, pie1b)

mean(pie1)

View(pie1)

net_pred <- qgraph(Fit$pairwise$wadj,

layout = 'spring', labels = labels, nodeNames = names,

border.width=1.5, border.color="black",

groups = grouplabels, color = group_cols,

legend.cex=.30, label.cex = 1.1, GLratio = 1.8,

vsize = 5, esize = 16, posCol = "darkblue", negCol = "red",

pie = pie1, pieBorder = 0.25, cut = 0,

filetype = "pdf", filename = "net_pred")

MgmModel <- estimateNetwork(data = data, default = "mgm", type = type, level = level, criterion = "EBIC",

tuning = 0.5, weighted = TRUE, binarySign = TRUE,

scale = TRUE, verbose = TRUE)

cor(vech(Fit$pairwise$wadj), vech(abs(MgmModel$graph)))

####Bootstrapping####

boot1 <- bootnet(MgmModel, nBoots = 1000, nCores = 6, type = "nonparametric", computeCentrality = TRUE)

save(boot1, file = "boot1.Rdata")

boot1F1 <- plot(boot1, labels = FALSE, order = "sample")

boot1F2 <- plot(boot1, "edge", plot = "difference",onlyNonZero = TRUE, order = "sample")

boot1F3 <- plot(boot1, "strength", plot = "difference")

boot2 <- bootnet(MgmModel, nBoots = 1000, nCores = 6, type = "case")

save(boot2, file = "boot2.Rdata")

boot2F4 <- plot(boot2, "strength")

corStability(boot2)

####Stability and accuracy####

pdf("EdgeStability.pdf")

plot(boot1, labels=FALSE, order="sample")

dev.off()

pdf("CentrStability.pdf")

plot(boot2)

dev.off()

pdf("CentralityDifference.pdf")

plot(boot1, "strength", order="sample", labels=TRUE)

dev.off()

pdf("EdgeDifftest.pdf")

plot(boot1, "edge", plot = "difference", onlyNonZero = TRUE, order = "sample")

dev.off()

**Table 1.** The matrix of edge weights.


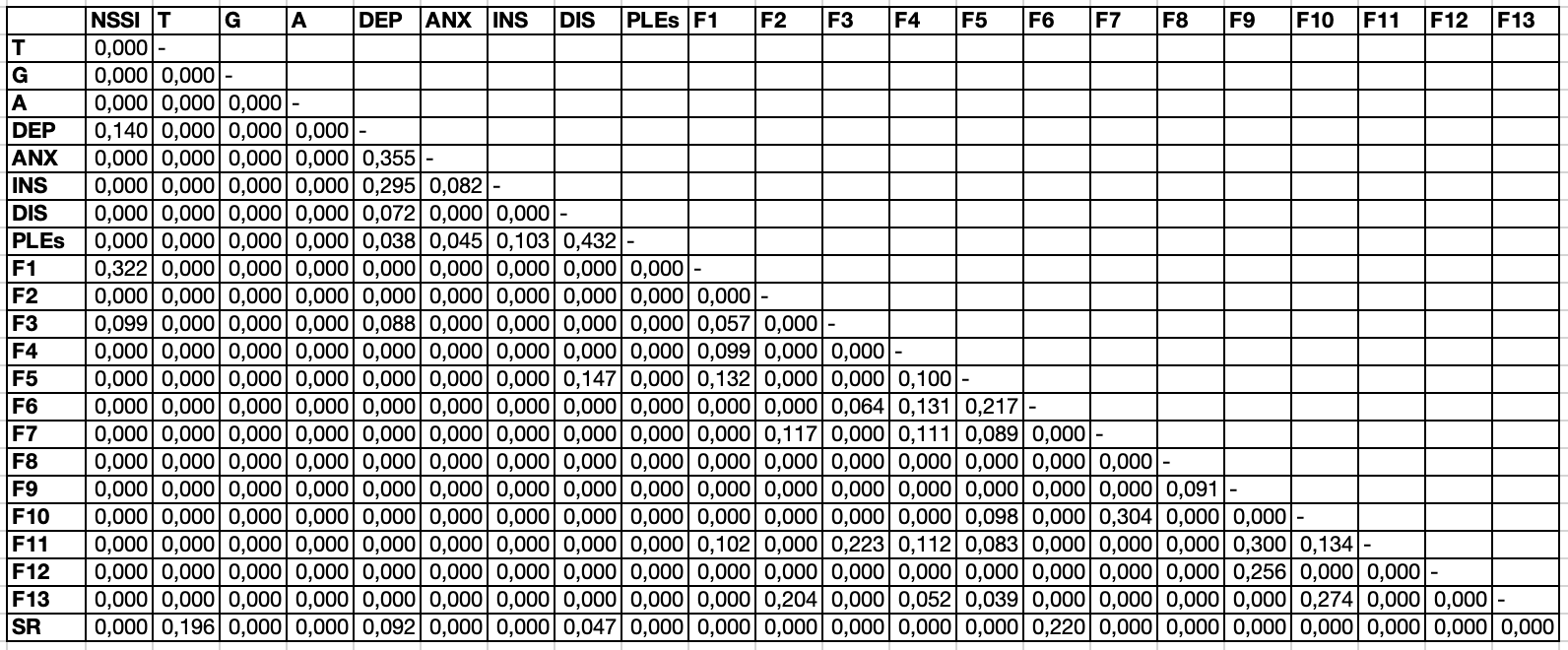


*Note:* A, age; ANX, anxiety symptoms; DEP, depressive symptoms; DIS, dissociation symptoms; F1, affect regulation; F2, interpersonal boundaries; F3; self-punishment; F4, self-care; F5, anti-dissociation; F6, anti-suicide; F7, sensation seeking; F8, peer bonding; F9, interpersonal influence; F10, toughness; F11, marking distress; F12, revenge; F13, autonomy; G, gender; INS; insomnia; NSSI, persistent non-suicidal self-injury; PLEs, psychotic-like experiences; SR, suicide risk

**Table 2.** Node predictabilities.

| **Node** | **Predictability** |
| --- | --- |
| NSSI | 0.345 |
| T | 0.000 |
| G | 0.000 |
| A | 0.000 |
| DEP | 0.489 |
| ANX | 0.338 |
| INS | 0.306 |
| DIS | 0.427 |
| PLEs | 0.420 |
| F1 | 0.292 |
| F2 | 0.179 |
| F3 | 0.288 |
| F4 | 0.333 |
| F5 | 0.441 |
| F6 | 0.343 |
| F7 | 0.341 |
| F8 | 0.026 |
| F9 | 0.288 |
| F10 | 0.419 |
| F11 | 0.433 |
| F12 | 0.162 |
| F13 | 0.328 |
| SR | 0.261 |

*Note:* A, age; ANX, anxiety symptoms; DEP, depressive symptoms; DIS, dissociation symptoms; F1, affect regulation; F2, interpersonal boundaries; F3; self-punishment; F4, self-care; F5, anti-dissociation; F6, anti-suicide; F7, sensation seeking; F8, peer bonding; F9, interpersonal influence; F10, toughness; F11, marking distress; F12, revenge; F13, autonomy; G, gender; INS; insomnia; NSSI, persistent non-suicidal self-injury; PLEs, psychotic-like experiences; SR, suicide risk

**Figure 1.** Bootstrapped differences between edge weights. Black boxes refer to significant differences between edge weights.


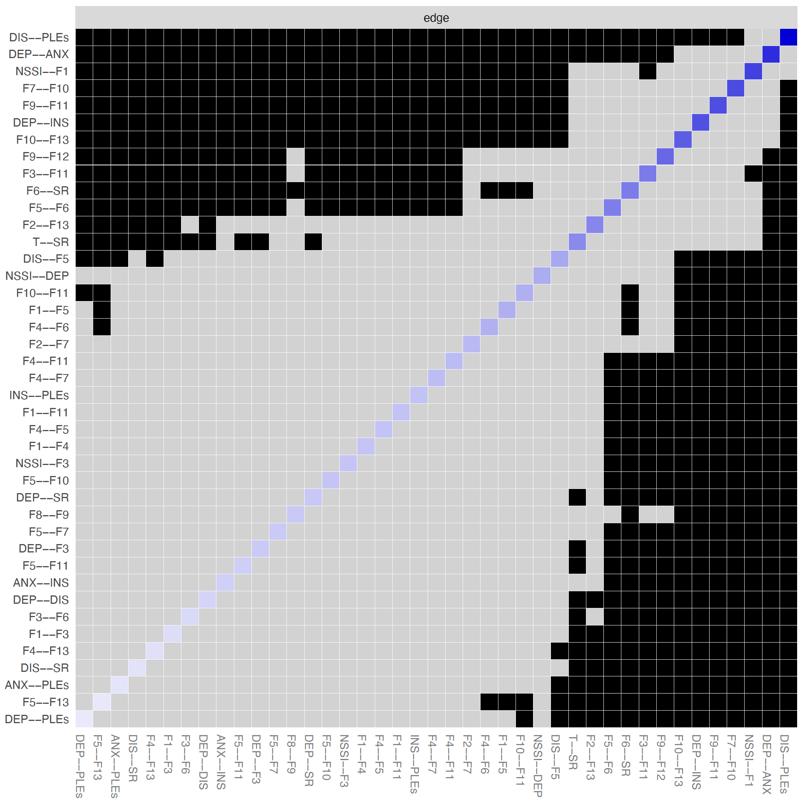


*Note:* A, age; ANX, anxiety symptoms; DEP, depressive symptoms; DIS, dissociation symptoms; F1, affect regulation; F2, interpersonal boundaries; F3; self-punishment; F4, self-care; F5, anti-dissociation; F6, anti-suicide; F7, sensation seeking; F8, peer bonding; F9, interpersonal influence; F10, toughness; F11, marking distress; F12, revenge; F13, autonomy; G, gender; INS; insomnia; NSSI, persistent non-suicidal self-injury; PLEs, psychotic-like experiences; SR, suicide risk

**Figure 2.** Stability of the strength centrality index. The red line shows changes in the strength centrality index after removing various proportions of data.

**Figure 3.** Bootstrapped 95% confidence intervals of edge weights. The sample values are illustrated with red lines. The bootstrapped 95% confidence intervals are illustrated within the grey area.


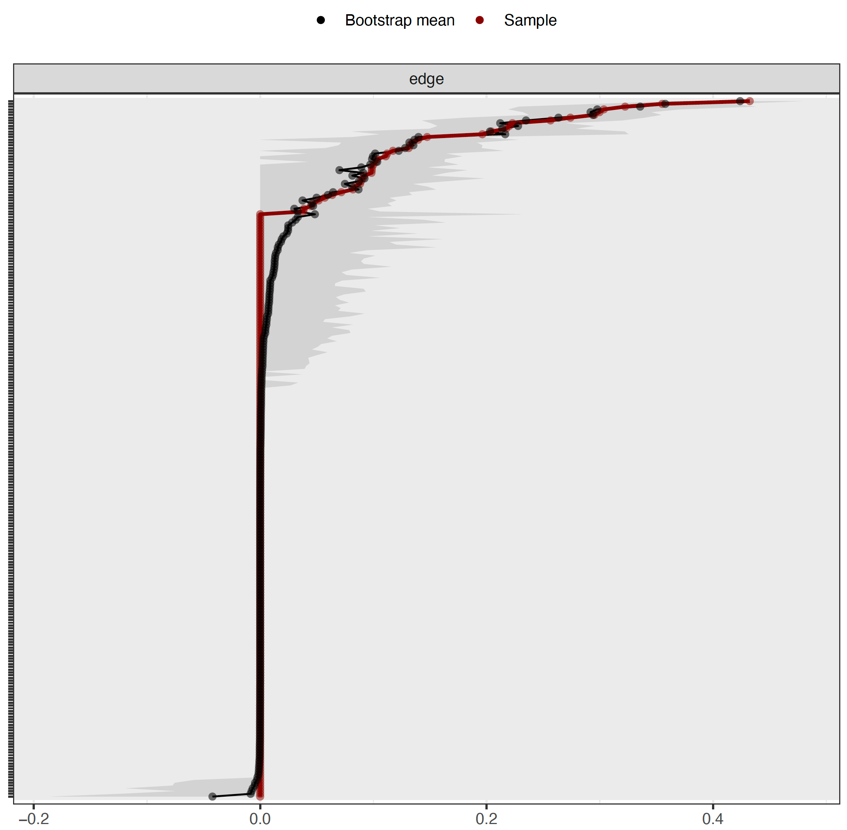

Supplement: Supplementary file 1 [file Table1.docx]
